# Pastoralists as Optimal Foragers? Reoccupation and Site Selection in the Deserts of Post-Soviet Kazakhstan

**DOI:** 10.1007/s10745-016-9870-5

**Published:** 2016-12-24

**Authors:** S. Robinson, C. Kerven, R. Behnke, K. Kushenov, E. J. Milner-Gulland

**Affiliations:** 1 Imperial College London, Silwood Park, Buckhurst Road, Ascot, Berks, SL5 7PY UK; 2 La Cousteille, 09400 Saurat, France; 3 Centre for Livestock and Veterinary Research, Dzhandosov Str. 31, Almaty, Kazakhstan 480035; 4 Department of Zoology, University of Oxford, South Parks Road, Oxford, OX1 3PS UK

**Keywords:** Pastoralism, Kazakhstan, Optimal foraging, Central place, Grazing

## Abstract

This study explores the drivers of site selection amongst livestock owners under conditions of increasing animal numbers following a low point in the 1990s. Our major goal was to understand whether livestock owners are acting as ‘optimal foragers,’ targeting areas of highest forage availability as they colonise previously empty areas. The results presented here suggest that they do not. Initially, distance from home settlement was the dominant determinant of site occupancy, with closer sites occupied earlier regardless of other characteristics. Some owners remained on depleted vegetation for longer than would be predicted under conditions of optimal foraging, indicating that distance-related costs constrained resource matching. In the latter period, increases in livestock wealth encouraged the occupation of distant sites exhibiting higher vegetation density and water quality, but some owners still occupied highly depleted sites. Improved transport and water supply infrastructure are needed if pastoralists are to optimise resource use across the landscape.

**Electronic supplementary material:**

The online version of this article (doi:10.1007/s10745-016-9870-5) contains supplementary material, which is available to authorized users.

## Introduction

Extensive pastoralism is a key livelihood strategy in arid regions where few other forms of land use are feasible (Thornton *et al.* 2002), and relies on the ability of animals to exploit spatial and temporal variation in forage resources, traditionally achieved through mobile animal husbandry systems. Yet globally livestock mobility is declining (Boone *et al.* 2008; Dong *et al.* 2011). Kazakhstan is no exception to this pattern and has suffered from a particularly extreme contraction in livestock mobility (Kerven *et al.* 2006) following the collapse of collective agriculture and a 75% drop in livestock numbers associated with the end of the Soviet Union (Behnke 2003). Economic conditions remain difficult - the costs of moving livestock now fall on individual households rather than large institutions, leading to loss of economies of scale. Although Kazakhstan has the fifth largest grazing resource in the world (FAOSTAT 2015), it has been estimated that only around 30% is currently grazed (Tazhibaev *et al.* 2014).

Livestock numbers are now increasing. By 2013, total livestock numbers (in sheep equivalents) had risen by 80% from their nadir in 1999, but still lie at around half of Soviet-era inventories (FAOSTAT 2015). Whilst this increase has not, in the few cases documented, brought about a full scale return to mobile pastoralism, some migrations including high mountain pastures have persisted (Ferret 2013) and re-colonization of abandoned desert areas is now occurring in some areas  (Kerven *et al.* 2016b). This is not the result of state planning, but of individual decision making. The Kazakh government would like to encourage the increased use of remote pastures, seeing these as a way of reducing supplementary feed costs (Ministry of Agriculture of the Republic of Kazakhstan 2013, 2014). The country as a whole suffers from a winter feed deficit (Government of Kazakhstan and the World Bank 2004) and in semi-arid areas, which comprise 80% of total grazing lands, supplementary fodder cannot be grown. It is here, particularly in key wintering areas, that optimal use of natural forage resources would bring the greatest benefits.

Our study area is one of the most important winter pastures in Kazakhstan and access brings significant economic benefits through a reduction in winter feed costs (Milner-Gulland *et al.* 2006). It is thus important to understand what is driving, and constraining, the process of re-colonization of such key pastures. The second issue of interest is pasture degradation. This is of concern in Central Asia because, although animal numbers are now low, mobility has declined, leading to high localised stocking densities with severe impact on soils and vegetation (Alimaev *et al.* 2008; Robinson 2016).

Most grazing systems in Kazakhstan contain multiple vegetation types, and these pastures are used at different seasons (Zhambakin 1995). Our study system is no exception and consists of four seasonal pasture types located in different ecological zones. Large scale movement between these zones is discussed elsewhere (Kerven *et al.* 2016a; Robinson *et al.* 2016). Here, we investigate the re-colonization of well-based sites by livestock herds and flocks across a large desert pasture in one of these ecological zones. The case constitutes an unusual natural experiment in which people retracted from a fully exploited landscape to concentrate their herds around settlements and then moved back out over a period of decades. One similar case is the re-colonisation of the Longone flood plain in Cameroon, in which re-flooding resulted in an influx of pastoralists, shown by the authors to occur roughly in proportion to increasing grazing resources (Scholte *et al.* 2006). In our case, pastoralists started from a central point (the settlement) from which they re-colonized sites defined by availability of water. We explore the factors underlying site selection by observing this re-colonization experiment over time. Given the related phenomena of pasture overstocking and abandonment that have been documented in Kazakhstan, we are particularly interested in whether herders are ‘optimal foragers’,  targeting areas of highest forage availability as they colonise previously empty areas. In order to understand this process, we use theoretical frameworks from the discipline of ecology, which predict how animals make decisions about where to forage in space and time.

### Optimal Foraging

It has long been suggested that animals primarily concerned with maximising their food intake will behave as ‘optimal foragers’. In an unconstrained space within which resources are patchy and travel costs are negligible, these foragers will firstly target resources of highest value (resource density compared to landscape averages). They will leave a patch when the marginal intake rate drops to the average for the habitat. This type of *individual-level* behaviour is captured in the marginal value theorem (MVT) (Charnov 1976): The richer the occupied patch is compared to the landscape average, the longer the patch residence time should be.

In an equilibrium environment, in which all patches are occupied, optimal foraging results in an ideal free distribution, which predicts that the ratio of individuals between two foraging sites will match the ratio of resources in those two sites, i.e., a matching of foragers and forage resources at the *population level* (Fretwell and Lucas 1970).

We are interested in whether our human agents respond to forage-related factors as the primary criteria for site selection in order to maximise food intake of their livestock, or whether other factors such as water availability or costs related to distance from the home settlement may be more important. The predictions of MVT are thus a convenient starting point from which to explore the factors determining herder decision making.

**Fig. 1:**
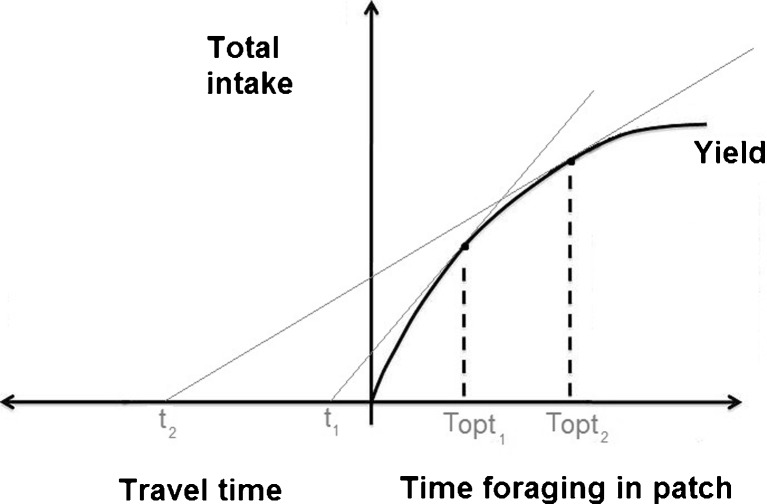
Operation of the classic marginal value theorem. As animals forage for longer in a given patch, intake is assumed to increase at a decreasing rate. The optimal residency time within a patch (T_opt_) depends on the relationship between travel time and the slope of the curve relating intake to foraging time. Where the marginal rate of intake (slope of the curve) is equal to the slope of the cost (travel time between patches), it is optimal for the animal to leave for the next patch. Therefore when travel time is longer (t2) the animal should spend longer in the patch than when the travel time is shorter (t1). Adapted from Charnov (1976) and De Boer and Prins (1989)

Bringing costs of accessing patches into the equation changes the predicted behaviour. For example, as travel time between patches increases, the time spent in a given patch is predicted to increase, because the costs of leaving are higher (Fig. 1). Such a relationship would suggest that greater travel times would lead to higher depletion of resources as animals spend more time on patches.

If there are two sites, one rich in resources but further from the settlement, and one poor in resources but close by, then two processes are operating to retain animals in the richer patch. As well as higher travel costs leading to higher residency time, the higher initial resource density means that the site will be grazed for longer before it reaches the landscape average resource density. Thus, foragers will travel further (bear more costs) for a richer patch where higher initial resource density compensates for the time travelled.

This theory helps us to investigate resource depletion and the potential for pasture degradation, because resource density metrics (e.g., vegetation cover or NDVI) are often easier to measure than forage intake by animals. For this reason, giving up density (GUD), the resource density at which animals leave a patch, is often measured in studies of MVT as an indicator of the intake rate at which patches are left (Brown 1988). For this study, GUD is interesting in itself – because very low GUDs may be associated with high levels of resource depletion and pasture degradation.

Key MVT predictions have been validated in domestic animals under controlled conditions (Laca *et al.* 1993; Utsumi *et al.* 2009), in wildlife (Astrom *et al.* 1990; Fryxell *et al.* 2004), and in human foragers such as hunter gatherers and fruit pickers (Hill *et al.* 1987; Wolfe 2013). As predicted, these authors found that total intake rate decreases as distance between patches increases, and that patch residence time increases with patch size (a proxy for resource density) and with distance between patches. De Boer and Prins (1989) found that these predictions also partially apply to herders of domestic animals, reporting that herders tended to evaluate trade-offs between travel time, foraging time and intake over the day rather than on a patch by patch basis, but that over a longer time frame they did optimise forage intake within the limits imposed by the need to return to the homestead at night. This brings up issues of spatial and temporal scale and of the effects of having a central place to return to, both of which we will now examine in more detail.

### Scale

Bailey *et al.* (1996) have defined six scales at which the foraging behaviour of large herbivores may be studied: bite, feeding station, patch, feeding site, camp, and home range. Most studies of herbivore foraging behaviour are conducted at the lower end of this scale, over minutes or hours and between individual patches of vegetation. It is only recently that studies have been up-scaled to look at seasonal movements over landscapes (Owen-Smith *et al.* 2010). Most studies at the upper end concern wildlife; few deal with herded livestock under human decision making.

Studies of foraging amongst domestic livestock in semi-arid environments often concern herds or flocks congregating around central places, to which they return at night and which represent water or shelter, rather than food resources. This results in the generation of a piosphere or grazing gradient around the central point (Lange 1969). Most studies concerning the foraging patterns of domestic animals (herded or not) concern *daily* movements around these central points, corresponding to the camp level (Coppolillo 2000; De Boer and Prins 1989; Moritz *et al.* 2010; Rozen-Rechels *et al.* 2015; Shrader *et al.* 2012; Turner *et al.* 2005).

This study looks at site selection, not around camps, but between multiple camps distributed at distances of several kilometres across a total area of hundreds of square kilometres, from year to year. We are therefore working at the landscape scale – or Bailey *et al*.’s “home range.” Only a few studies have mapped or modelled herded livestock at this scale (Boone *et al.* 2008; McCarthy 2007; Moritz *et al.* 2014; Scholte *et al.* 2006; Vanselow *et al.* 2012). Some (e.g., Moritz *et al.* 2014) have tested for an ideal free distribution (IFD) at the population level. An IFD would be the predicted result of optimal foraging decisions by individual herders in an equilibrium environment where the ratio of gain to cost is the same across the landscape. But our expanding non-equilibrium environment means that an ideal free distribution is unlikely to have yet emerged. We are thus able to expose the processes involved in site selection over time in a way that is not possible in an equilibrium situation. Our study system is also rather different to those to which MVT is usually applied. Its defining characteristics are common in pastoral systems but rarely appear in ecological studies on wildlife (Table 1).

**Table 1:** Defining characteristics of the study site relevant to studies of foraging

Currency to be optimised	Profit
Spatial scale	Landscape or home range
Patch definition	Wells, to which livestock are restricted at a radius of around 6 km
Temporal scale	Multi-annual: the set of wells to be used is decided in advance on an annual basis. Grazing periods at wells usually last months
Central place effects	The distance from home villages to wells is more likely to influence site selection than distance between wells used by the same livestock owner
Costs	Most costs co-vary with distance. Metabolic costs are incurred once a year during migration. Costs of bringing supplies are incurred many times over grazing period
Group foraging effects	Larger herd sizes likely to reduce costs of movement *per animal*
Predation	Possibly a factor, but not of high significance between sites within the desert

The study area has an added advantage in that ‘patches’ are self-defining. MVT describes resource consumption in a patchy habitat. But patchiness is a relatively difficult concept to pin down in grassland habitats in which resources may be continuously distributed in space and time (Fryxell 2008). In our case, each well and its associated grazing radius may be construed as a discrete ‘patch.’ This makes it conceptually simple to define both patch occupation and patch abandonment as presence and absence at wells.

Wells may be used for the whole year or just part of the year. Livestock owners may use several wells in the same year, or one alone, but the set of wells to be used over the year is decided *in advance* because rental contracts for sites must be made at the beginning of the year or earlier. Thus, decision making events occur in advance and the temporal scale at which site selection must be studied is *multi-annual*.

### Central Place Considerations

Our theory so far would predict that total intake increases with environmental quality but that the GUD is the same for all patches, unless costs are higher at some patches than at others. Although we are not addressing grazing patterns *within* piospheres, our system also exhibits certain central place characteristics. In terms of primary family residence, livestock owners are based in three settlements. Those using several wells over the year may try to ensure that these are located close to each other, but it seems likely that the primary consideration will be distance from the home settlement. Within a landscape we would expect to find a relationship between distance and total intake if there is a relationship between distance and environmental quality – e.g., if forage is denser further out from central points. In our system, patch quality is indeed likely to be positively correlated with distance from settlements.

Large distance-related costs in a central place foraging scenario could delay leaving times of patches closer to the settlement and decrease the GUD of those patches, despite denser patches further out, contrary to the expectations of MVT when there is no central patch to return to. This would result in a situation much like that of a piosphere, in which closer sites would become depleted and distant sites would become relatively richer, resulting in positive correlations between distance from settlement, total intake, and environmental quality.

Close to home, GUD and forage intake rates when the patch is left are low because the short distances compensate for the high missed opportunity costs (MOC) of the better forage in distant patches. Once MOC become larger than distance-related costs, it is worth changing patches. Herd size is likely to offset distance by reducing costs through economies of scale. We use this model to set up our hypotheses.

### Hypotheses

The non-equilibrium situation means that there is unlikely to be a direct relationship between resource density and stocking rate, but the situation of expansion across a single zone is suitable to test the following two hypotheses, which relate to the beginning of the expansion process and would hold if gain is strongly correlated with resource density and if travel-related costs are low:

(i) Resource density may be higher at occupied sites than unoccupied sites if herders are targeting higher vegetation density under optimal foraging conditions and have not yet depleted patches to the landscape average.
(ii) Considering the expanding nature of site selection over time and ignoring the cost of travel, we predict that sites occupied earlier have a higher resource density than those occupied later, or at least this would have been true during the early stages of re-occupation.

If travel costs are much higher than the MOC of not moving, then livestock will deplete resources to far below the average level for the landscape, leading to very low GUDs. In such cases, we would expect to see that hypotheses (i) and (ii) would be refuted. In addition we would expect to observe the following conditions:

(iii) Higher levels of relative depletion (not absolute resource density) outwards from wells at occupied sites than unoccupied sites;
(iv) Lower depletion levels at more recently occupied sites than sites occupied for several years or occupied and then abandoned;
(v) An overriding role for distance in the system: sites close to our putative central point, the village, would be colonized first regardless of forage availability considerations;
(vi) Amongst occupied sites and over time we would expect to see a positive relationship between vegetation density and distance from villages, as the ratio of gain to cost should start to equalise over the landscape. We would also find negative relationships between forage depletion and distance as forage is depleted closer to villages;
(vii) Lastly, if herd sizes have a mitigating effect on costs, we would expect to see a positive relationship between herd size and distance from the central point.

These hypotheses may be tested straightforwardly in an environment in which patch quality (before grazing) is distributed randomly. Yet patch quality distribution is not random, but exhibits gradients across the landscape. Moreover, forage density is certainly not the only site characteristic of possible interest to herders. Water quality (salinity) and accessibility (depth) have been shown to influence site selection, whilst snow cover and relief affect vegetation and site accessibility (Kerven *et al.* 2016b) and also exhibit gradients across the study area. We also explore how the spatial distribution of these additional factors influences site selection.

## Methods

We take an interdisciplinary approach using qualitative data from interviews with herders, local officials and livestock professionals, quantitative data on stocking metrics and a set of bio-physical variables to examine each hypothesis.

### The Study Area

**Fig. 2:**
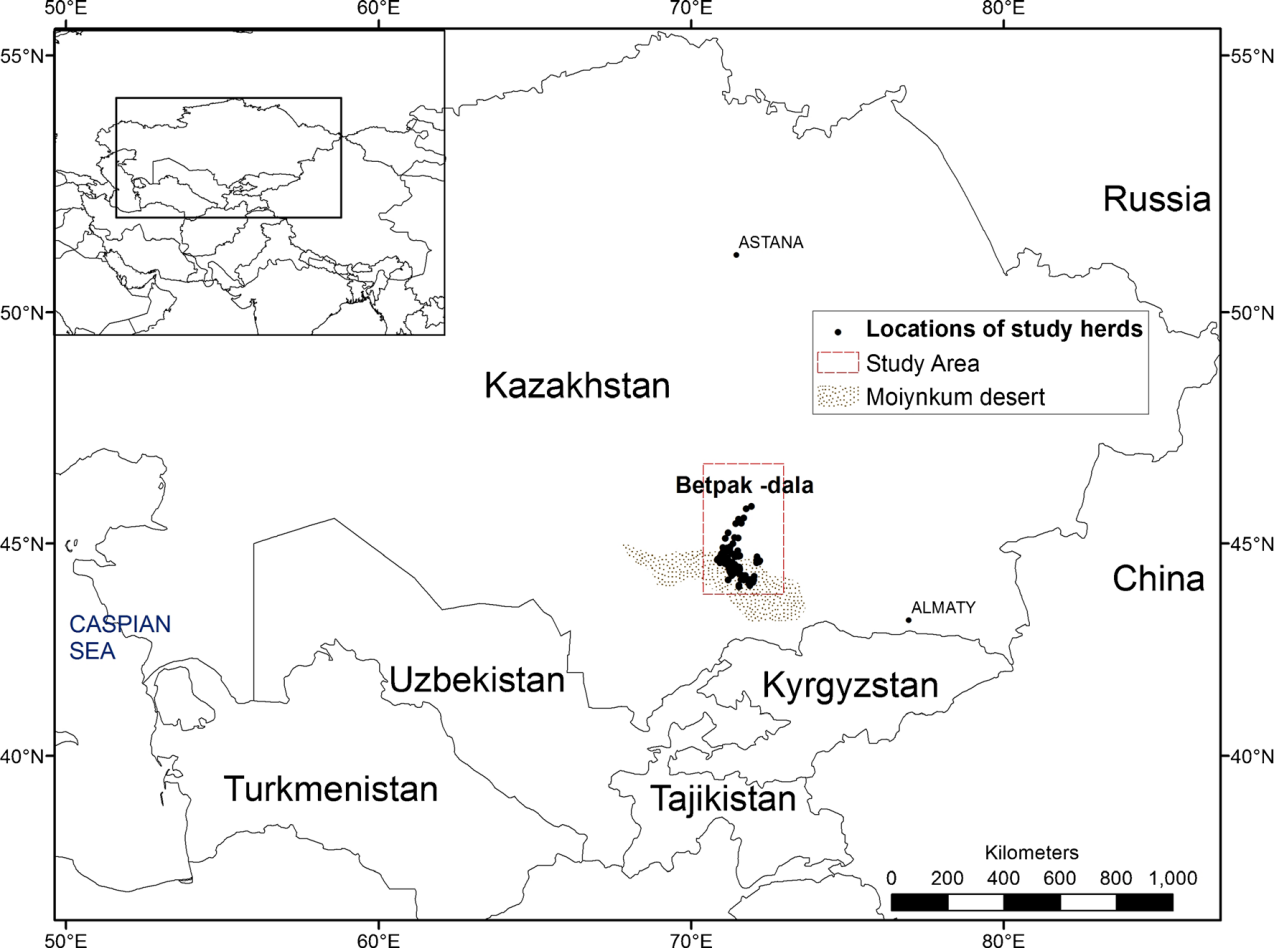
Location of Moiynkum desert in Kazakhstan

**Fig. 3:**
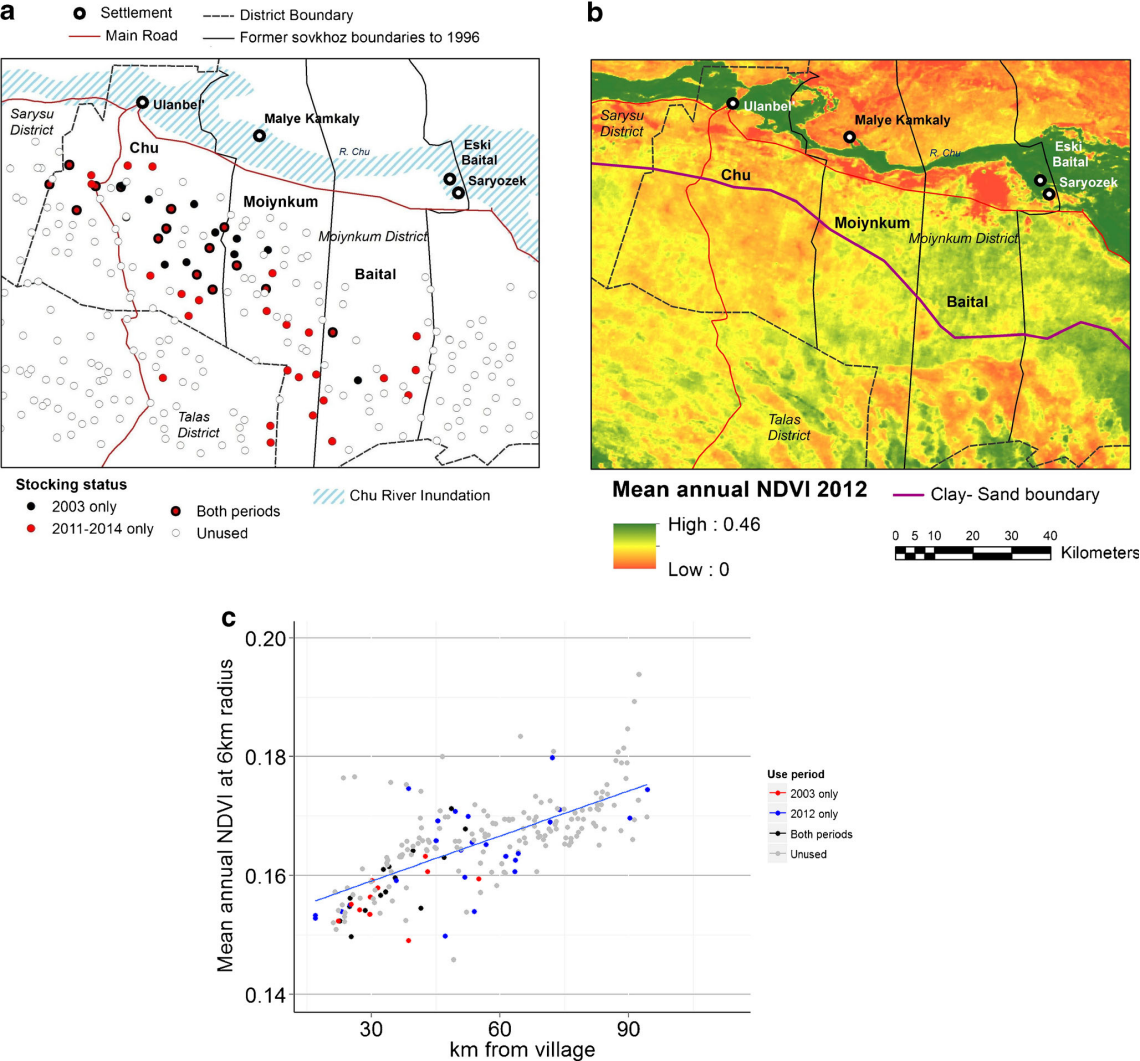
Site occupation and resource density in the Moiynkum desert, (**a**) Well distribution and occupation in 2003 and 2011–2014 (**b**) Mean NDVI in 2012 across the study area (**c**) Relationship between mean annual NDVI at 6km radius from wells and distance from Chu valley settlements at used and unused sites: b_1_ = 2.676e-04 (se = 1.893e-05); R^2^ = 0.49; *p* < 0.0001

The Moiynkum is a sandy desert located in Dzhambul province of south-central Kazakhstan just south of the river Chu (Fig. 2). It originally constituted the wintering pasture of a much larger migratory system in which livestock spent the summer over 300 km north of the river Chu (Fig. 3a), along which the human population and associated settlements are based. Today, although some livestock are moved a reduced distance to the north into the clay desert of Betpak-dala (Fig. 2), most owners now base their livestock along the river Chu or in the Moiynkum desert, and use one or both of these areas over the year.

The study area includes three former *sovkhoz* (Soviet era state collective farms), Chu, Moiynkum and Baital, whose populations were concentrated in the settlements of Ulan bel’, Malye Kamkale, and Sary Ozek respectively (Fig. 3a). Chu and Moiynkum have today been merged to form a single administrative sub-district (Chu), whilst Baital now forms half of the adjacent Karabuget sub-district to the east.

By the 1980s, the Moiynkum desert was highly degraded, with severe deflation around wells (Bizhanova 1999; Dzhanpeisov *et al.* 1990). Today, livestock numbers are around only 15–20% of Soviet-era levels, and pasture condition is likely to have recovered to some extent. Since the collapse of the *sovkhoz*, some herders have established long-term bases around wells. In spring and summer, users may move animals from these bases to temporary encampments in open access areas along the Chu river and in Betpak-dala. By contrast, the bases in the Moiynkum are located on formally leased pasture and often have investments in infrastructure such as barns and houses (Kerven *et al.* 2016b). Base sites  may be occupied for many years, and yet site abandonment and re-occupation was also common during our study. The sample of occupied and unoccupied wells is large enough for basic statistical investigation but not for multi-variate analysis, so the role of resource density and other factors that possibly determine site selection was examined separately.

### Data Collection and Processing

#### Livestock Data

Formal questionnaire censuses were conducted with all livestock owners present in the study area in 2003 and 2012. The census asked owners to list pasture locations and water sources used by their large stock and small stock (separately) over 12 month periods starting from October 2002 and October 2011. Overall, 118 unique livestock owners were recorded. Of these, full livestock ownership information was available for 80 owners in 2003 and 84 in 2012 (58 appeared in both years). According to the census data, 49 owners used the Moiynkum desert at some period of the year in 2002-2003, and 30 in 2011–2012 (note that due to the practice of collective herding several owners may use the same sites, just as one owner may use more than one site). The information on stock type, numbers, water source, and monthly location name were entered into a database. Numbers were converted to standard livestock units (LU) often used by pasture scientists in Central Asia and similar to cattle-sheep ratios used on rangelands elsewhere (USDA/NRCS 2003): one sheep or goat is one LU; one cow, horse or camel is five LU. These units were multiplied by the number of days at the location during the relevant time period to give a single comparable figure for each location expressed in ‘livestock unit years’ (LUY). This metric is equivalent to one head of sheep grazing at a location for one year.

Open-ended interviews were conducted with 97 individuals (many interviewed more than once) during five fieldwork periods between 2012 and 2014. Interviewees included livestock owners, shepherds, village mayors, veterinary officers, former employees of the state livestock farm, and employees of the state forestry department. Interviews were used to gather livestock owners’ perceptions on site selection and to identify important variables affecting decision-making. The results are discussed in detail elsewhere (Kerven *et al.* 2016b) and summarised here briefly in relation to our hypotheses and findings.

During interviews conducted in 2013 and in spring 2014 a number of additional occupied wells were recorded, including some which were newly occupied and a small number missed during the original census, but exact dates of use and livestock numbers were not recorded. Thus, livestock presence data were available for wells occupied in 2002–03 and between the end of 2011 and the start of 2014 (concerning mainly the years 2012 and 2013), whilst density (stocking rate) information was available on a monthly basis for two single year periods running from the months of October 2002 and 2011.

Concerning the exact locations of used sites, many of the surveys and interviews were conducted at grazing sites, recorded by GPS. Livestock owners and other knowledgeable interviewees identified additional used locations on a set of 1:50,000 Soviet era topographical maps. The majority of these sites were then visited by vehicle with key informants and recorded by GPS. The locations of those sites that could not be visited were digitised directly from the georeferenced topographical maps on which all wells are shown, many with names. Most such locations were verified on the maps by more than one interviewee. All locations, with their coordinates, were inputted into a relational database, thus providing exact locations for every site listed in the livestock survey table.

Livestock presences were recorded as locations having any stock presence over the year, whilst absences were defined as locations having wells that existed in the Soviet era, but which were not recorded as occupied either in 2002–3, or between 2011 and 2014. These may be considered potential sites, and were digitised from the topographic maps (which  show all wells and outbuildings existing during the 1980s, after which no new infrastructure was built).

Over the past few years, livestock owners whose animals are based at settlements along the Chu have started to move them to wells outside former *sovkhoz* boundaries. For this reason the maximum extent of our analysis was set to cover an area stretching up to 30 km from these boundaries. Within this area, of the total of 445 occupied and potential sites, 282 had infrastructure (buildings, barns) and were considered to be major sites likely to have been used as bases in the past; others consisted of isolated wells and may have been occupied for shorter periods on a temporary basis. Both sets of sites were used in analysis for comparison, but the set of sites with infrastructure was considered to be the most conservative estimate of potential livestock locations existing during the later Soviet period (Fig. 3a). Not all of these have working water supply facilities today, many having been destroyed, dried up or become too expensive to exploit.

In addition to the set of 282 potential sites, a number of geographical subsets were used during analysis including the restricted set of 130 sites^1^ within former sovkhoz boundaries, where we can be virtually certain that we have no false absences - i.e., any users of these wells from outside were also recorded and included in the census. Outside these boundaries, false absences are possible as the knowledge of our informants regarding these outlying areas may have been incomplete, and some wells may have been used for herds from other administrative areas. Livestock from Sary Ozek make little use of the desert and so the dynamic process of site occupation examined in this study principally concerns Chu and Moiynkum, corresponding to modern Chu sub-district. Therefore, some analyses were also performed on this subset of sites alone, concerning 102 potential sites. Most of these have been re-occupied from Ulan bel’ village as Malye Kamkale is virtually abandoned.

Overall, livestock were present at 26 locations in 2002/2003 and 40 during the full fieldwork period of 2011–2014 (of which 37 were within former *sovkhoz* boundaries and 30 in Chu sub-district). Twenty-seven of these were recorded with full livestock number information for our specific census period October 2011–October 2012. The rest were recorded as having livestock presence at some point between October 2011 and April 2014. These data represent two snapshots of well occupation in a complex 20 year-long process of well occupation and abandonment. Our data do not include information on the exact dates and length of occupation periods between the two study periods. However, the year of first occupation since the collapse of the *sovkhoz* was obtained for a subset of 29 wells for more detailed investigation of site sequencing. The sets of occupied sites are different in the two time periods, altering the choice of unoccupied sites. This is addressed by explicitly comparing site characteristics between sites occupied in 2011–2014 alone, occupied in 2003 and then abandoned, and occupied in both periods.

#### Resource Density

The remote-sensing derived Normalised Difference Vegetation Index (NDVI) is used worldwide as an indicator of green biomass (Pettorelli *et al.* 2011). We employed a gapless 250 m 16 day NDVI dataset generated from the MODIS MOD13Q1 product (Vuolo *et al.* 2012) for the years 2001–2012, from which we extracted annual averages both over the entire period and for the years 2003 and 2012 alone (see supplementary materials 1). Average NDVI values were extracted around each location (usually where the livestock were watered or spent the night) at a radius of 6 km, shown to be the typical extent of grazing impact around desert wells in Kazakhstan (Karnieli *et al.* 2008). Vegetation composition was also investigated using a vegetation map (Fig. S1 supplementary materials 2) generated from a MODIS-based classification (see supplementary materials 1).

We have not attempted to match the season of use of the Moiynkum desert to NDVI values at time of use. In the migratory system as a whole movements are partly influenced by seasonal variations in vegetation availability which occur between vegetation zones (Robinson *et al.* 2016). However, within the Moiynkum vegetation types are more homogeneous. These sites constitute primary bases from which other, more temporary moves may be made. By integrating NDVI over the year, we are looking at the overall attractiveness of a site as a ‘base’ regardless of the season in which it was used at the time of the study.

#### Resource Depletion

In many arid areas of Central Asia, forage is relatively homogeneous around wells; wells and shelter are located in the same place and forage availability tends to increase, and grazing pressure to decrease, away from this single central point (Alimaev *et al.* 2008; Kanchaev *et al.* 2003; Nechaeva *et al.* 1979). Differences between NDVI at 0-2 km and 3-5 km from wells, or NDVI gradients, were measured using the annual average NDVI data for 2003 and 2012. These gradients may be interpreted as a measure of resource depletion and have been shown to be closely related to stocking density metrics elsewhere in the region (Behnke *et al.* 2016).

#### Water Availability

Both well depth and salinity have been found to influence site selection, the influence of depth acting through the cost of pumping (Kerven *et al.* 2016b). Water quality was estimated by livestock owners as good, average, or salty for a subset of 41 wells, and interviewees also provided depth information for 39 wells.

#### Other Variables Affecting Site Selection

It was assumed that travel costs to occupied sites from home settlements were proportional to distance, which was measured using the ARCMAP point distance tool. Products from the MODIS instrument were processed to produce multi-annual snow cover images at a resolution of 5 degrees (see supplementary materials 1). Relief, in the form of sand dunes, was mapped at a 30 m resolution using visual enhancement and interpretation of an ASTER Digital Elevation Model (DEM).

#### Statistical Analysis

Relationships between distance from settlement and NDVI, date of first occupation and NDVI, stocking rate and NDVI gradient, and well depth and first date of occupation were explored by ordinary least squares linear regression. Differences in mean NDVI, NDVI gradient, distance from settlement, well depth and snow cover between locations with stock presence and absence were assessed using Welch’s t-test; differences in these variables between the four site stocking categories (used in 2003 alone, 2011–14 alone, both periods or unused) were assessed using one-way ANOVA. All statistical analyses were carried out in R (R Core Team 2014). Image processing and analysis were carried out using the Raster package for R (Hijmans and van Etten 2013) and ARCGIS (ESRI 2011).

## Results

### Well and Livestock Distributions in the Moiynkum Desert

Wells with infrastructure in the sand desert portion of our three *sovkhoz* territories were located on average 3.5 km apart (well below the radius at which animals graze) so the area was essentially ‘full up’ with potential sites in Soviet times. The distribution of wells (with infrastructure) mapped within all *sovkhoz* boundaries is close to a distributed regular pattern (*n* = 130, nearest neighbour ratio = 1.7, z-score = 1.72, *p* = 0.08) and within Chu sub-district alone follows a highly distributed pattern (*n* = 102, nearest neighbour ratio = 1.14, z-score = 2.7, *p* < 0.01). The equivalent analysis using all wells reveals a random pattern, suggesting that a planned effort was made to regularly space sites with infrastructure and confirming that the presence of infrastructure might be the best metric to define absences (potential yet unoccupied sites). These findings also suggest that either underlying environmental factors did not influence well distribution, or that these factors do not display a spatial gradient or clustering pattern.

Today, large areas of the Moiynkum desert are unoccupied but the number of occupied sites has risen from 26 in 2002–3 to 40 in 2012–13, with 15 sites occupied in both periods. Some sites are occupied all year but others only seasonally. Seasonal use is now highly variable, ranging from one to four seasons rather than in spring/autumn (north of the desert) and winter (southern desert) which characterised the Soviet period (Fig. S2, supplementary materials 3).

### Characterising Resource Gradients across the Study Area

The occupation of new sites has occurred mostly to the south and east (Fig. 3a). There are large groups of sites, particularly to the south of Ulan Bel’ and northern parts of former *sovkhoz* Moiynkum, which are unoccupied. We discuss how forage resources, and other natural factors possibly influencing site selection, vary across the desert.

#### Vegetation and Soils

The north of the desert, which has clay takyr-like soils, has few wells because the ground water is salty. This area is covered by vegetation dominated by *Artemisia* and *Haloxylon* spp. (see vegetation type 1, Fig. S1, supplementary materials). This pasture makes poor grazing outside the autumn and only two occupied wells were located here. Further south, the soil becomes progressively sandier (Fig. 3b). Transitional areas dominated by *Ceratoides* (vegetation type 2 in Fig. S1) give way to vegetation dominated by shrubs such as *Calligonum*, *Haloxylon*, *Astragalus* spp. which, together with an understory including *Ceratoides*, *Kochia* and *Atriplex* spp., provide good forage all year round. By far the majority of wells are located in this vegetation type on sandy soils (south of the clay/sand border in Fig. 3b and corresponding to type 3 in Fig. S1), which is rather homogeneous, apart from areas affected by fire, visible as patches of low NDVI in the southeast of Fig. 3b. The increasingly sandy soils to the south present transport difficulties as deeper sand combined with higher dune size increase both fuel costs and the risk of vehicles becoming stuck, thus requiring heavier and more expensive transportation.

#### Biomass

NDVI appears to increase towards the east of the study area and in some parts of the southeast (Fig. 3b). This follows a precipitation gradient. Average annual precipitation ranges from 150 mm close to the Chu river to 180–200 mm in the central Moiynkum (Kurochkina and Osmanova 1973) and 294 mm in the south (Bizhanova and Kurochkina 1989). The existence of a biomass gradient independent of resource depletion by grazers is evident (Fig. 3c), which indicates a strong relationship between NDVI and distance (to the south) from settlements, not only for occupied sites, but also for sites unoccupied in either study period.

#### Water and Snow

Our sample of wells, representing those for which respondents had knowledge, exhibited decreasing salinity towards the south from settlements, a factor identified as important in site selection by herders (Kerven *et al.* 2016b and supplementary materials Table S1). Those wells having average or good quality water tend to be deeper close to Ulan bel’ and shallower to the south and east (Fig. S3). Thus, for a livestock owner based at Ulan bel’, although most nearby wells are relatively shallow, many are of poor quality, while the deeper good quality wells are expensive to use. Further away in former Moiynkum and Baital *sovkhozes*, those wells known to respondents tended to be both shallow and of good or average quality. These particularly attractive wells are now occupied by owners based at Sary Ozek and Malye Kamkale; others are occupied by owners from Ulan bel’ who have also tended to move greater distances in recent years. Despite higher precipitation, snow cover is also lower to the south and east, probably influenced by higher temperatures, particularly in early spring (Fig. S4, supplementary materials 5).

#### Distance

The role of distance from settlements as a constraint to site occupation is evident (Fig. 3a). More recently occupied sites appear to be located further away from settlements, a phenomenon even more exaggerated when we consider that in fact most expansion is from the settlement of Ulan bel’ alone. However, the expansion appears rather directional towards the south and east, so distance, whilst important, must interact with other factors.

We now turn to a more formal analysis of relationships between these factors and site occupation.

### Resource Density

Our first hypothesis was that occupied sites may be those with higher long term average resource density. We examined this by comparing average annual NDVI (2001–2012) at occupied and unoccupied wells. Taking livestock occupancy during the 2011–2014 period, there was no significant difference in mean NDVI values between the 37 occupied and 93 unoccupied locations within the three former *sovkhoz* boundaries at a 6 km radius from the well (*t* = 0.87, *p* = 0.39). But at a radius of 1 km, where grazing pressure is greatest, occupied sites had significantly *lower* NDVI (*t* = 2.46, *p* < 0.05). The 2003 set of occupied sites exhibits very strong differences in NDVI when compared with sites unoccupied at that time, even at 6 km radius from wells, with occupied sites having significantly lower NDVI (*n* = 130 wells of which 26 occupied; *t* = 4.2, *p* < 0.0005). These differences were also noted using NDVI in the year of use instead of 12 year annual average NDVI. Thus site selection decisions made in individual years are unlikely to depend on resource density characteristics in that specific year, and resource densities in a given year do not differ radically from long term averages.

**Fig. 4:**
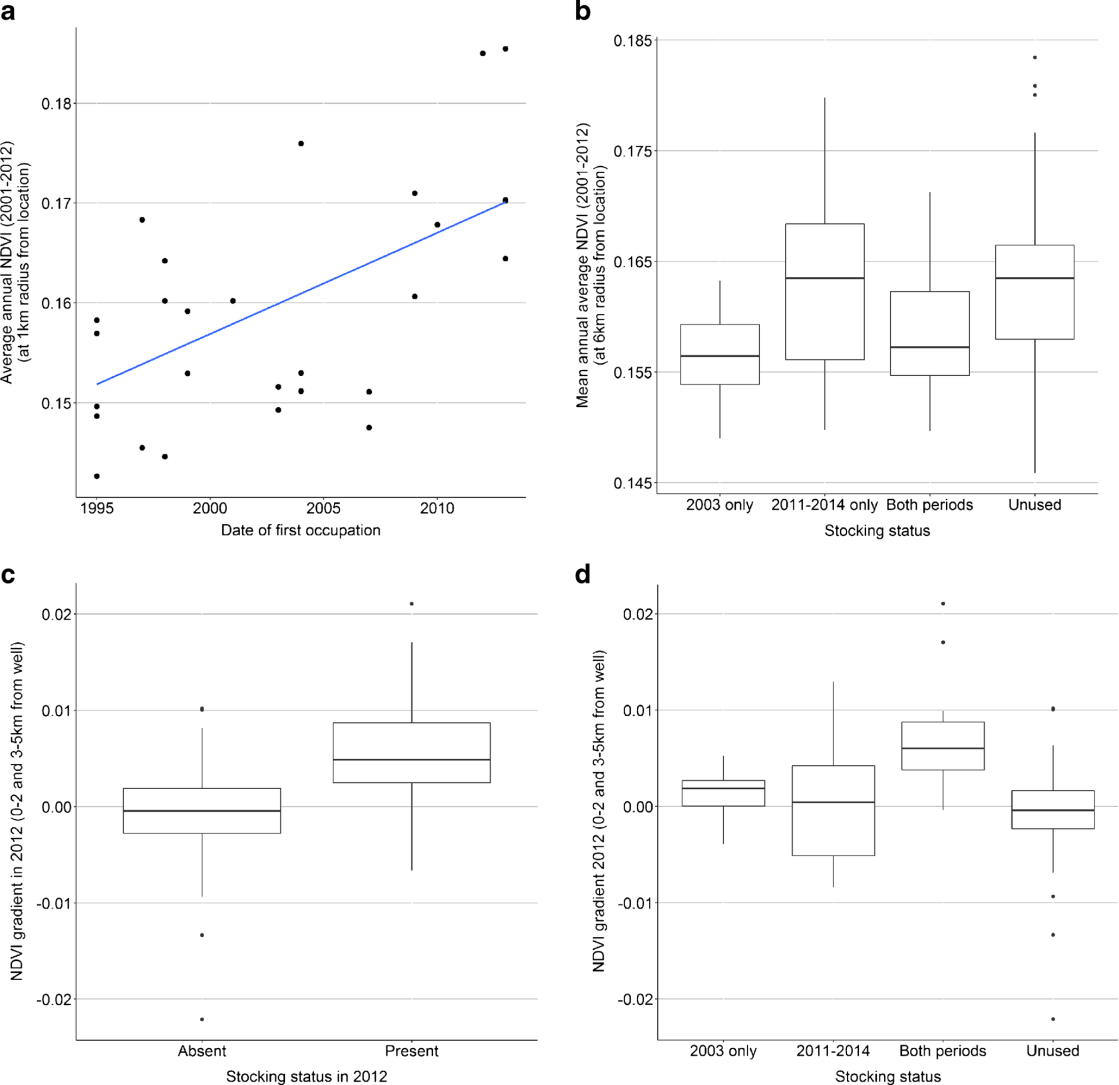
**a** Regression of annual average NDVI (2001–2012) against year of first re-occupation after collapse of the sovkhoz (*n* = 29): β_1_ = 0.001 (se = 0.00028); R^2^ = 0.327; *p* < 0.005. **b** NDVI at sites used in 2003, 2011–14, both periods and unused sites, within sovkhoz boundaries (*n* = 11, 22, 15, 82). There was a significant difference in group means (F = 4.4; *p* < 0.01). Post hoc comparisons using the Tukey HSD test indicate no difference between mean NDVI at sites used in 2011–14 and unused sites (*p* = 0.99); significant differences between unused sites and those used in 2003 (*p* < 0.05) and near significance between sites used in 2011–2014 and in 2003 alone (*p* = 0.064). A t-test on these latter two group means is significant: *t* = −3.1, *p* < 0.005. **c** 2012 NDVI gradient at occupied and unoccupied sites within former sovkhoz boundaries in 2011–2012 alone (census year only; present *n* = 27; absent *n* = 103). A positive gradient implies that NDVI increases out from the well (a measure of resource depletion); negative gradients imply that NDVI decreases out from the well. The difference in mean gradient is significant: *t* = −4.8, *p* < 0.0001. Taking all sites used from 2011 to 2014 (present *n* = 37, absent *n* = 93) the relationship is weaker as many of these were used for the first time *t* = −3.0, *p* < 0.005. The equivalent test for 2003 data (*n* = 26; 104) is significant: *t* = −4.1, *p* < 0.0005. **d** 2012 NDVI gradients at sites used in 2003, 2011–14, both periods and unused sites, within former sovkhoz boundaries (*n* = 11, 22, 15, 82). There was a significant difference between group means (F = 10.44; *P* < 0.0001). Post hoc comparisons using the Tukey HSD test indicated that the difference in mean NDVI gradient between newly occupied sites (2011–14) verses sites occupied in both periods is significant (*p* < 0.001); difference between sites occupied in 2003 alone and in 2011–14 alone is insignificant (*p* = 0.9); difference between sites used in both years and sites never used is significant (*p* < 0.0001). Note: boxplots shown here (and all subsequent boxplots) show median values (horizontal lines in box centre), and the 1st and 3rd quartiles (top and bottom of boxes). The whiskers extend to values of 1.5 x Q3-Q1. Outliers are shown as dots and represent values > Q1–1.5*IQR and < Q3 + 1.5*IQR where IQR = Q3 - Q1. a)

Our second hypothesis was that most resource dense sites would be occupied earlier. Here, we regressed annual average NDVI against year of first occupation of sites since the collapse of the *sovkhoz*. Whilst no relationships were evident at 6 km radius, a weak relationship did emerge closer to wells (Fig. 4a), but again the direction of the relationship is the inverse of that predicted by MVT, with those sites occupied first exhibiting *lower* NDVI than sites occupied later on.

Relationships between resource density and the order of site occupation were explored further by comparing NDVI at the sets of sites occupied in 2003 alone, those occupied in 2011–2014 alone, and sites occupied in both years (Fig. 4b). Sites occupied after 2003 (i.e., appearing in the 2011–2014 dataset only) have mean NDVI levels very close to those of unoccupied sites, whilst those occupied in 2003 and during both periods have much lower NDVI values.

The above analyses suggest that differences in resource density between occupied and unoccupied wells, both in space and over time, are related to vegetation depletion caused by grazing at various periods during the 12 years over which the NDVI data are averaged. In this scenario, our theory predicts that when travel costs are high, sites are occupied beyond the point at which the forage acquisition rate drops below the landscape average. In this case, site occupancy determines vegetation density rather than the reverse. Additional evidence for such a scenario is that NDVI differences between occupied and unoccupied sites in 2012 and 2003 are larger (with higher statistical significance) when the full set of sites with wells (not only those having infrastructure) are included in the analysis. Whilst wells with infrastructure are likely to have been occupied in the past, those without infrastructure may have been little or never occupied, thus amplifying the potential difference in accumulated grazing pressure between occupied and unoccupied sites. This also suggests that past grazing effects are detectable for long periods.

However, we have seen that there is also a north-south gradient in ‘natural’ vegetation density related to climatic factors rather than livestock grazing. Our most recently occupied sites, as well as many unoccupied sites, appear to be located further south. Thus we must examine not only absolute resource density, but relative depletion levels around wells in order to better understand the process behind the observed patterns.

### Resource Depletion

We now therefore turn to our hypotheses concerning NDVI gradients outwards from wells. As hypothesised, average NDVI gradients were steeper at occupied than at unoccupied sites in both 2003 and 2012 (Fig. 4c). Secondly, we examined average NDVI gradients in 2012, at sites with different occupation histories, to address the hypothesis that more recently occupied, or more briefly occupied, sites should exhibit less resource depletion. Within *sovkhoz* boundaries, sites occupied only in recent times (appearing in the 2011–2014 dataset alone) exhibited no discernible difference in NDVI gradient in 2012 from unoccupied sites, which, as would be expected, have a median gradient of around zero (Fig. 4d). Sites occupied in both periods exhibited high levels of depletion. The difference between sites occupied in both periods and those appearing only in the 2011–2014 dataset is particularly strong. However, depletion at sites occupied in 2003 but subsequently abandoned is slight, and mean gradients are statistically indistinguishable from those at sites occupied only recently (Fig. 4d). These results suggest that (i) the effects of depletion require several years of site occupation to become evident as NDVI gradients, and (ii) the impact of past grazing appears to decrease over the timescale at which we are working. We should remember here that NDVI gradients are not measuring the *quality* of grazing - weedy species may persist for many years after NDVI gradients are no longer detectable.

It should be noted that although we have found relationships between livestock presence and resource depletion, relationships were not found between actual livestock *density* (annualised stocking rate) and the extent or size of the gradient in either 2003 or 2012. This may be because stocking rates are not variable enough to cause detectable differences in depletion level.

Overall, at least by 2012, site occupation appears more likely to be causing resource depletion than to constitute a response to variability in vegetation density, consistent with the scenario that distance-related costs were the dominant factor determining site selection. However, although the MVT prediction that the most resource dense sites would be occupied first has not been shown to be correct, it is difficult to refute convincingly because we are essentially dealing with two temporal ‘snapshots.’ Our dataset does not have the fine temporal detail of occupation and abandonment dates which could perhaps have been used with associated NDVI data at dates of initial occupation to untangle these two factors. If relationships between resource density and date of first occupation do exist, they are most likely to be *negative,* as those sites occupied first are furthest north, where precipitation is lowest. Those sites occupied in 2003, but abandoned before the subsequent study period, have low NDVI gradients (little depletion) in 2012 (Fig. 4d), but also have low long-term average absolute NDVI values (Fig. 4b), suggesting that these sites may be generally resource-poor in a way not related to depletion alone. This may also explain why the difference between NDVI at occupied and unoccupied sites was so much greater for the 2003 dataset (significant at 6 km) than for the 2011–14 dataset (non-significant at 6 km).

A second prediction of classical MVT, that movement away from sites would occur once they had been grazed down to the landscape average resource density, appears not to be supported, at least for the 2003 dataset. Even in recent years, although some herders have recently colonised un-depleted sites, others have stayed on patches long past the GUD predicted by MVT*,* again supporting the idea that costs of movement are high. We now turn to hypothesis (v) concerning distance from the home settlement (Table 2).

**Table 3:** Results of hypothesis testing

№	Hypothesis	Results
(i)	Resource density is higher at used sites than at unused sites (IFD)	False in 2003 & 2012.
(ii)	Order of site occupation by individual herders since the collapse of the sovkhoz is positively related to resource density (IFD)	False
(iii)	Resource depletion is higher at sites where livestock are present than at those where they are absent.	True in 2003 & 2012
(iv)	Resource depletion is lower at more recently occupied sites than (a) sites used for many years and (b) sites used and then abandoned.	(a) True
		(b) False
(v)	Within specified spatial boundaries, sites at which livestock are present are closer to settlements than sites from which they are absent.	2003: True
		2012: False
(vi)	For the later dataset we expect: (a) positive relationship between forage density and distance from villages; (b) negative relationship between forage depletion and distance from settlements	(a) True
		(b) Inconclusive
(vii)	If larger herd sizes mitigate costs of movement we would expect to find positive relationships between herd size and distance from settlements.	False in 2012

### Distance

According to this hypothesis, if other factors were equal then we would expect the sites closest to villages to be settled first. This question is explored for Ulan bel’ sub-district (former Chu and Moiynkum sovkhoz) alone, as so few stock from Sary Ozek are located outside the settlement. In this sub-district most livestock are coming from the settlement of Ulan bel’. Results confirm that indeed occupied sites were significantly closer to Ulan bel’ than unoccupied sites in 2003 (Table 2). However, by 2012 there was no significant difference between occupied and unoccupied sites. It thus appears that either suitable sites closer to Ulan bel’ are all occupied or a reduced importance of travel costs (e.g., lower fuel costs or higher returns from meat sales) means that factors other than distance are increasingly important in site selection. Certainly 11 of the sites occupied in 2003 close to Ulan bel’ were subsequently abandoned, and there are still many empty wells throughout the study area, so reduced travel costs may be more important. No relationships between stocking rate and distance from Ulan bel’ were found.

**Table 2:** Distance from Ulan bel’ and livestock presence and absence (for sites within the boundaries of Chu sub-district).

Year	n (absence)	n (presence)	Mean distance, km (absence)	Mean distance, km (presence)	t	p
2003	78	24	49.6	37.2	4.3	<0.0001
2012	72	30	46.6	46.9	-0.081	ns (0.9359)

Yet the impact of distance on site selection still had consequences during the later study period. If we compare sites occupied in 2011–14 with the theoretical larger universe of unoccupied sites across the wider Moiynkum zone, then NDVI values at sites occupied in 2011–14 are *significantly* lower than those of other sites, even at a radius of 6 km (242 wells of which 40 occupied; *t* = 4.2, df = 68.12, *p* < 0.001). This is because there are many unoccupied sites in the highly vegetated south of the sandy massif. These sites are potentially available and indeed a few were occupied by 2013.

If costs increase with distance then we would expect GUD to be lower closer to settlements and higher farther away, as the ratio of cost to gain starts to equalise over the landscape (hypothesis vi). By the end of our study period, we would thus expect to see a gradient of resource density outwards from settlements at sites occupied during our study period, but not at unoccupied sites. However, this hypothesis cannot be confirmed due to the natural north-south increase in resource density evident at both occupied and unoccupied sites (Fig. 2c). It is in 2013 and within *sovkhoz* boundaries alone that there is most likely to be equalising of cost-gain ratio. We did indeed find a strong positive relationship between distance from the home settlement and NDVI at sites that had been occupied at any time during the study period (*n* = 48; β_1_ = 7.2e-04 (se = 6.0e-05); R^2^ = 0.76, *p* < 0.0001). However, there is also a strong relationship between NDVI and distance for sites that were not occupied in either period (*n* = 82; β_1_ = 7.6e-04 (se = 7.3e-05); R^2^ = 0.57; *p* < 0.0001), albeit with a lower slope. We would also expect to find negative relationships between forage depletion and distance from settlement at occupied sites, as forage is depleted closer to settlements (again calculating distances as above). Here although a slightly negative relationship was observed, it is extremely weak (b_1_ = −9.6e-05 (se = 4.5e-05); R^2^ = 0.08, *p* < 0.05).

Lastly, we expected that if economies of scale could mitigate travel costs, herd size should be positively related to distance from the village. We explored the relationship between both average herd size over the months each site was occupied, and annualised stocking rate (in LUY) and distance from village for the year 2012. No relationships were apparent here for either variable, however the sample size of sites for which stocking information was available was small (*n* = 27). The difference in herd size between sites occupied in 2012 and sites occupied in both 2003 and 2012 was also non-significant.

### Other Factors

Other factors that interviews with herders and local professionals suggested were important determinants of the cost or attractiveness of particular sites were water quality (saline or not), depth and type of the well (which may affect extraction cost), topography (which may affect travel cost), and snow cover (which may impede grazing in winter but provide important moisture in spring) (see Kerven *et al.* 2016b). As we have seen, the water quality of wells varies with distance from settlements, such that less saline wells are further away (Table S1 in supplementary materials). Of the 41 wells for which salinity scores were given, 24 of the 25 wells listed as good or average were occupied between 2011 and 2014, whilst the 16 saline wells were occupied and unoccupied in equal proportion. In 2003 there was no difference in the proportion of occupied saline and non-saline wells, suggesting that selectivity for water quality may have increased over time. Likewise, no relationship was detected between water quality and date of first occupation for our subset of 29 wells for which year of first occupation after collapse of the *sovkhoz* was available.

**Fig. 5:**
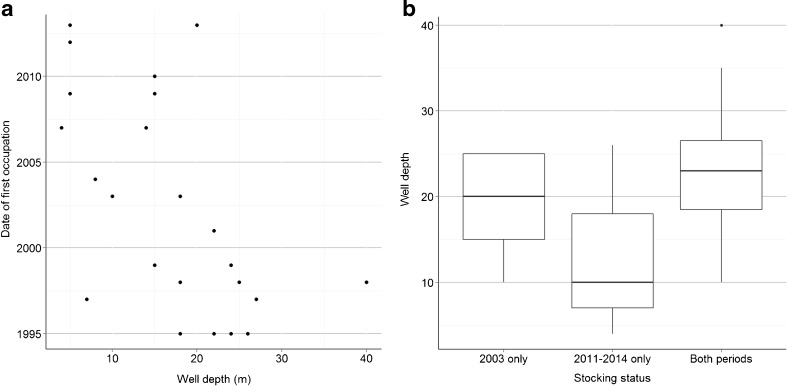
**a** Well depth and year of first occupation (*n* = 23): β_1=_-0.8494 (se = 0.2522); R^2^ = 0.35, *p* < 0.005. Note: two very deep outliers (80 m) have been removed. **b** Well depth and use period category. Sites used in 2003 only: mean = 19 m, *n* = 5. In 2011–2014 only: mean = 13 m, *n* = 17. In both periods: mean = 24 m; *n* = 14. T-test for difference in mean depths of wells used in 2003 or both years verses those used in 2011–2014 alone (*n* = 19; 17, means =22.7 m & 12.7 m) is significant: *t* = 3.8, *p* < 0.001. Sites unused in either period not included as sample size *n* = 3 is too small

There were no significant differences in well depth between occupied and unoccupied wells in 2011–14, but the sample size of unoccupied wells (8) for which depth data were available was small. In 2003, occupied wells were significantly deeper (*n* = 39; unoccupied *n* = 20, occupied *n* = 19; means =12.5 m and 22.7 m; *t* = −4.06, *p* < 0.0005). The possible conclusion that shallower wells tend to be occupied later on is supported by weak relationships between well depth and both date of first occupation and use period category (Fig. 5).

These data are circumstantial at best – the set of wells for which information could be gathered represents that for which informants had knowledge, and the limit of our research resources. Most data for wells unoccupied in 2003 concern those which were in fact occupied later on (hence existence of knowledge about these wells in 2012–2014 interviews). There are very few data for wells unoccupied in either period. This is because herders are unsure about the status of wells that have not been visited for a long time. However, interviews do confirm that pumping costs increase with well depth and that water quality is an important pull factor in site selection (Kerven *et al.* 2016b).

Lastly, we examined the effect of water source type on livestock presence. The Soviet-era grazing plan of Ulan bel’ shows all major shaft wells and bore holes (*n* = 58). Sites having only bore holes may be the least likely to be occupied today as they require the most powerful pumps. Indeed, of the 25 sites at which bore holes alone are indicated, only five were occupied in either 2003 or 2011–14 (of which three have been occupied since 2012). Of the 33 sites with shaft wells or both types, 18 (54%) were occupied (*X* ^2^ = 7.1, *p* < 0.005). Most unoccupied sites lie directly south of Ulan bel’ nearer to the metalled road. The fact that most of them are bore holes may explain why those sites, despite being easily accessed, are rarely occupied (Fig. 2a, Fig. S5, supplementary materials 6). In this area, livestock owners also mentioned transport difficulties related to sand depth and dune height (Kerven *et al.* 2016b). Sandy soils may increase travel costs related to the occupation of all southern wells, but those in the south west may be particularly hard to reach (supplementary materials 6).

Historically, one of the values of the Moiynkum desert as a winter grazing area lay in its relatively low snow cover. Today, although many sites are also occupied in other seasons (Fig. S2, supplementary materials 3), winter is still a key grazing period. Snow cover as measured by satellite imagery is significantly lower at more recently occupied sites (2011–14 only) compared to those occupied in 2003 alone, but the absolute mean differences are very small (Fig. S6, supplementary materials 7). Moreover, more snow may be positive for spring growth, thus in interviews some herders considered snow to be an important factor determining site selection whilst others did not, depending on season of use. This factor is also likely to be confounded with geographical location, which as we have seen, correlates with other factors varying along the same north-south gradient.

To summarise, it appears that over time, herders have been increasingly targeting shallower and less saline wells in areas with higher vegetation density.

## Discussion and Conclusions

A major goal of this paper was to understand whether livestock owners are acting as ‘optimal foragers,’ targeting areas of highest forage availability as they colonise previously empty areas. The results presented here suggest that they do not follow the classical MVT (hypotheses i and ii, Table 3). Rather, it appears that travel costs are potentially critical determinants of site occupation, meaning that animals stay longer at sites than would be expected under classical MVT, leading to resource depletion (hypotheses iii–vi, Table 3). This is despite the continued existence of many unoccupied sites in the study area.

We found that occupied sites have significantly lower vegetation density and higher NDVI gradients than unoccupied sites. Such a pattern may seem rather predictable in semi-arid systems in which livestock are constrained to water points. However, even unoccupied sites may have continued to exhibit depletion from heavy use in the Soviet era; at the start of the study the time scale needed for more recent grazing activity to become detectable was unknown - the effects of grazing in 2012 were not detectable in the NDVI data at 6 km radius.

Livestock have stayed at these sites longer than would be predicted under conditions of optimal foraging, and have thus grazed vegetation to levels lower than would be expected if they were just responding to differences in forage availability between sites. GUDs are low, suggesting that costs of movement are high. These costs tend to co-vary with distance from settlements and were clearly the overriding factor determining site selection, at least during in the initial phase of expansion. Distance imposes costs not only in the one-off movement of livestock at the start of the grazing period, but in the many trips back and forth for supplies and family visits (Kerven *et al.* 2006).

Secondly, sites occupied later on have on average, both higher vegetation densities and lower depletion levels than those occupied at the start of our study period. Thus over time, depletion may indeed be driving livestock to new sites with high vegetation density, located further from the central point of departure. However, these sites do not just exhibit high levels of un-depleted vegetation. As we noted, they also tend to host sweet and shallow wells. Well salinity and snow cover vary along the same spatial gradient as vegetation density so it is difficult to untangle their relative importance. Herders’ perceptions suggest that water-related factors are important in site selection and abandonment, with depletion also a notable ‘push factor.’ Absolute vegetation density was less commonly given as a ‘pull factor.’ Whichever predominates, livestock owners have clearly selected more attractive sites in 2012 than in 2003.

The operation of the MVT, predicting movement from depleted patches to new patches once costs and gains equalise, is only one of the processes occurring here. The other is that the ratio of costs to profit is changing over time, thus the depressing effect of cost on GUD may also lessen over time. This is indeed illustrated by the declining influence of distance on site selection between our two study periods.

Part of this decrease in costs may be related to an increase in livestock numbers, which operates to decrease economies of scale (Kerven *et al.* 2004, 2006, 2008). Although we have shown in previous work that herd size is strongly related to the number of ecological zones occupied by migratory livestock (Robinson *et al.* 2016), we have not been able to demonstrate relationships between herd size and distance within the single zone studied here. However, the number of small stock in our study area roughly doubled from 2003 to 2012. This increase has translated both into a greater number of flocks using the Moiynkum desert, and to an increase in median flock size belonging to individuals, by around 70% over our study period (Kerven *et al*. 2016b). Higher meat prices and lower pumping costs have also contributed to general increased wealth of herders in recent years.

This may have relaxed constraints originally imposed by distance, allowing livestock owners to target better sites. Such a proposition is supported by the fact that the colonisation of increasing numbers of sites has been accompanied by a simultaneous process of site abandonment, as evidenced by the group of wells occupied in 2003 alone, and suggesting that the factors favouring the initial choice of certain sites subsequently changed over time. Resource depletion at early-occupied sites, a suite of positive characteristics of unoccupied sites, and a decrease in distance-related costs may have all led to the observed patterns of site occupation over time.

Despite these processes, and the recent colonisation of many new sites with low depletion and high vegetation density, a large proportion of sites occupied in 2003 continue to be occupied today, and this set of sites in particular still exhibits strong depletion – indicating very low GUDs and high cost to gain ratios. In order to use sites in the Moiynkum, considerable investments must be made to restore wells, winter houses and barns. Even if owners were able to “sell” these assets, the costs of rebuilding them at a new and more distant sites is likely to be high. Although recent trends suggest movement out of depleted areas, these have not yet resulted in optimal foraging patterns across the landscape. It remains to be seen whether such patterns will emerge as livestock numbers rise still further.

The use of the MVT to structure our investigations has been useful because it allows us to weigh up the opposing ‘pull factors’ of resource density (and other attractive site characteristics) against the constraints imposed by distance. The relative outcome of these two opposing forces is measured in our specific factor of interest – resource depletion, interpreted in the theory through the concept of GUD. This approach has allowed us to show empirically over a large area what range scientists in Kazakhstan have observed since independence – an uneven use of rangeland resources leading to a combination of localised overuse and abandonment (Alimaev *et al.* 2008; Rachkovskaya and Bragina 2012).

This study has shown the dominance of economic considerations over forage-related issues in herders’ decisions about how best to use resources, in contrast to a pastoral system in Africa which has been subject to similar analyses (Scholte *et al.* 2006). Such a situation is likely to be the case in pastoral systems that are increasingly subject to sedentarisation of households in a central place and reliance on mechanised transport. In terms of policy, the Kazakh government could intervene in a number of ways to reduce the high costs related to low GUDs and high levels of resource depletion. These include improving transport links, increasing the attractiveness of distant sites by repairing wells and other infrastructure and ensuring that administrative and other transaction costs of land access are as low as possible. It could also act to incentivise seasonal movement out of the Moiynkum desert, as much of the strongest depletion comes from livestock kept in the desert in the summer. Overall, the government needs to weigh the cost efficiency of such policies, in terms of improved access to natural forage, against its current policies which favour the intensification of livestock production.

## Electronic supplementary material

ESM 1 (PDF 1486 kb)

## Footnotes

1 All potential site numbers concern those with infrastructure on the maps.
