# Supplementary material for: Pastoralists as Optimal Foragers? Reoccupation and Site Selection in the Deserts of Post-Soviet Kazakhstan
Source: Hum Ecol Interdiscip J. 2016 Dec 24;45(1):5–21. doi: 10.1007/s10745-016-9870-5 (PMC5323497; doi:10.1007/s10745-016-9870-5)
Supplement: Supplementary file 1 — (PDF 1486 kb) [file 10745_2016_9870_MOESM1_ESM.pdf]

Supplementary materials

1. Additional detail on methods

Processing of MODIS NDVI and snow cover data: The complete dataset of 23 16 day NDVI composites (2001-2012) for the study site was downloaded and processed (including cropping and re-projection). The dataset was already filtered and smoothed, but further errors were removed by setting all negative values to zero (Hird and McDermid, 2009). Mean annual NDVI was taken as the average of all 23 images. The NDVI data generated included annual averages for the entire period from 2001-2012 and for 2003 and 2012 alone. In case snow cover affected relative average annual NDVI readings between sites, growing season NDVI was also extracted and the same analyses presented here were performed using this dataset. However, as the results were little different from those using annual NDVI, these are not presented here. The NASA Snow Cover product: MOD10CM (Hall et al., 2006) was obtained from http://modis-snow-ice.gsfc.nasa.gov/.

Mapping vegetation type: Forage maps of the three sovkhoz from 1977 and 1978 were available at a scale of 1:50,000. These maps were produced by the State Institute for Land Management known as Giprozem (Ministry of Agriculture of the Kazakh SSR, 1969) based on extensive fieldwork campaigns. A detailed methodology concerning the combination of these maps with an existing Landsat classification and recent ground truth data, to produce a MODIS-based classification of the study area is to be published elsewhere and is described also in Robinson et al. (2016).

2. Vegetation type in the study area

Figure S1. Vegetation types in the Moiynkum desert

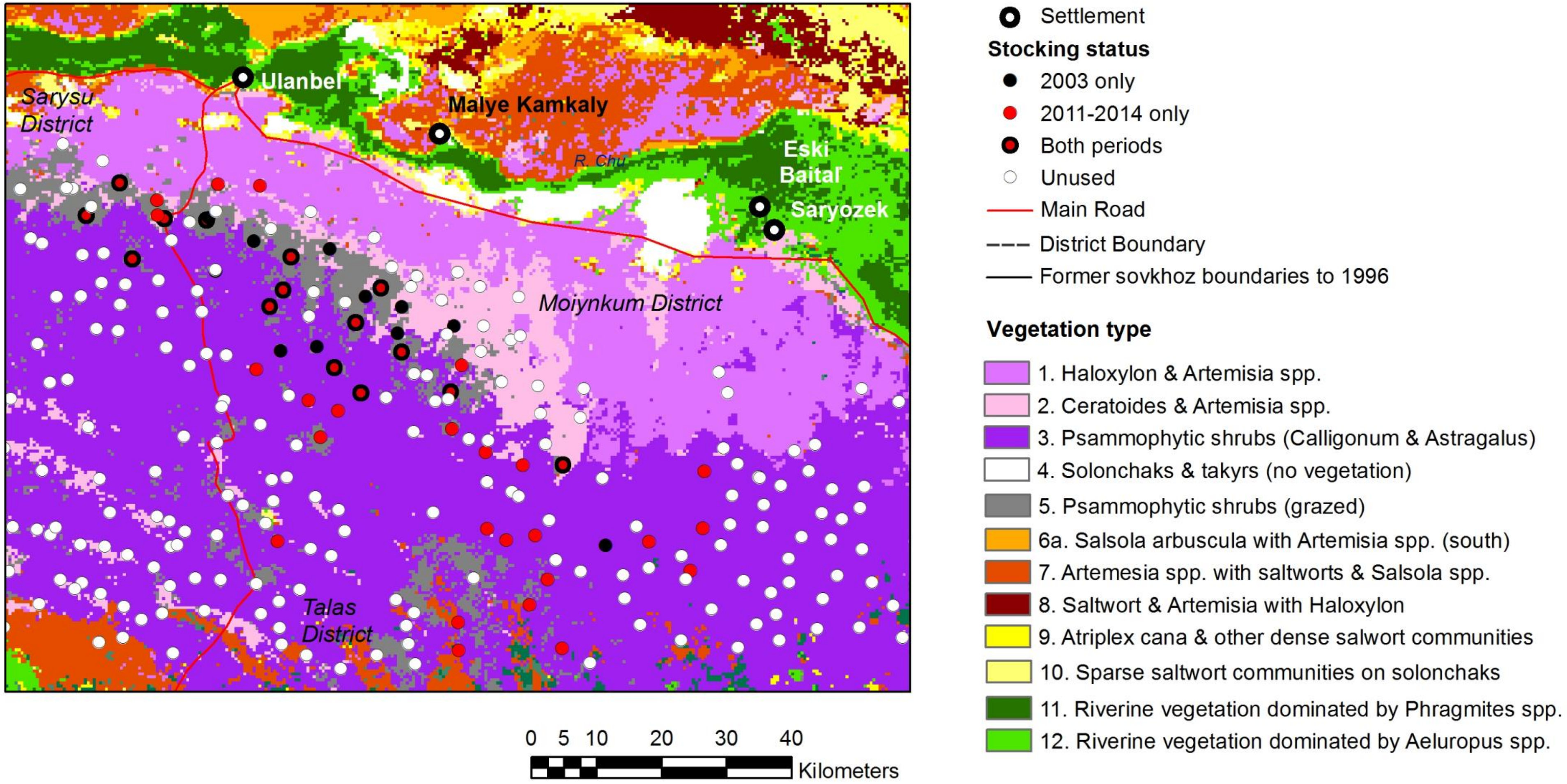

3. Season of use at wells in the Moiykum sands

Figure S2. Changes in season of use at wells in the Moiynkum sands belonging to former Chu sovkhoz, *(a) in the Soviet period and (b) during the period of the 2011-2012 livestock census.*

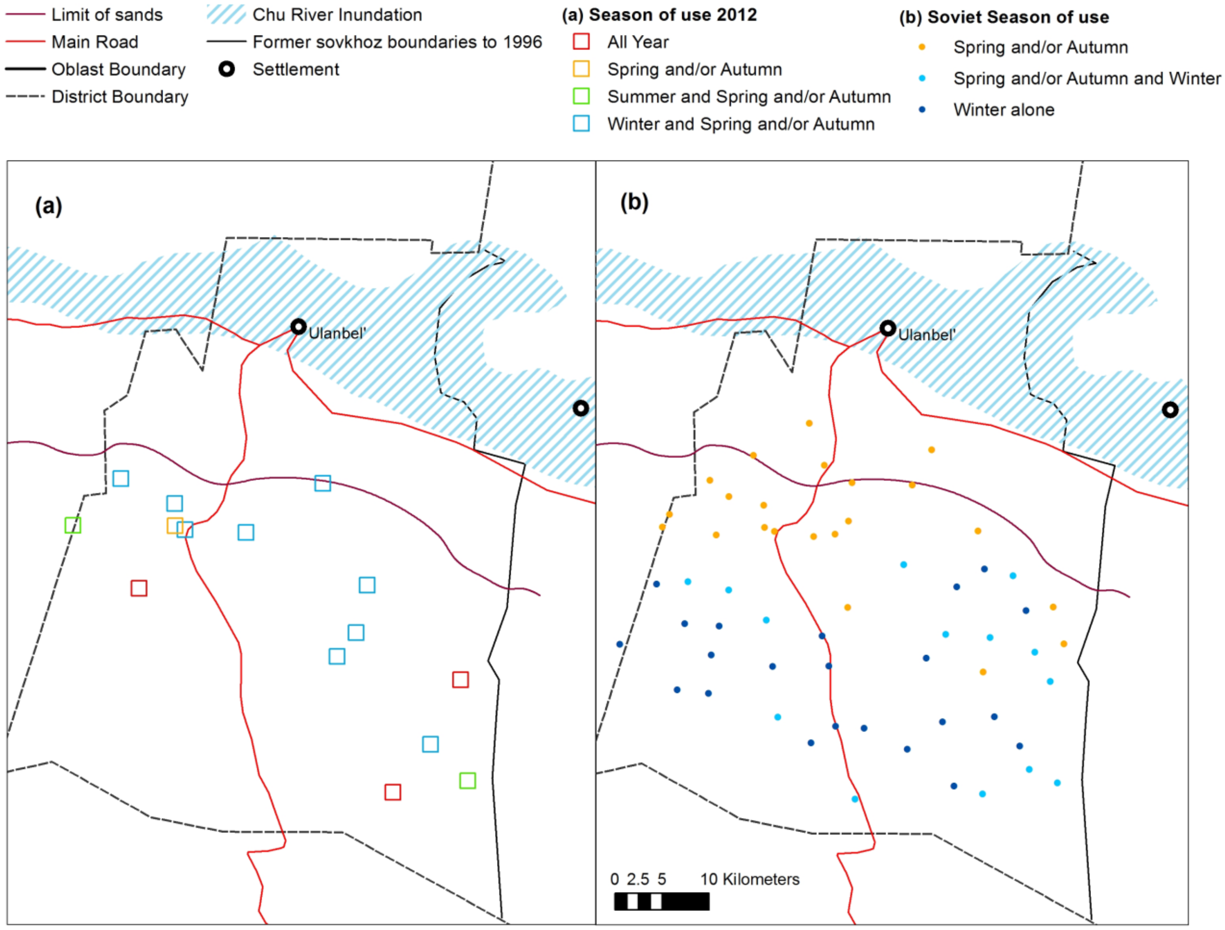

4. Distance, well depth and water quality

Table S1. Water quality and distance from settlements (difference in mean distance between good quality wells and the merged group of average and salty wells (n=17; 24) : t=2.09, df=34.209, p<0.05. Distances calculated from each well to the settlement with which it was associated in the Soviet era.

Water quality	Good	Salty	Average	Salty/Average
Number of wells	17	16	8	24
Mean distance from settlement (km)	45	33	39	35

Figure S3. Well depth, water quality and distance from Ulan bel'. Note: 'Talas' refers to areas of the desert in Talas district, south of our study area – see Fig. S1 above.

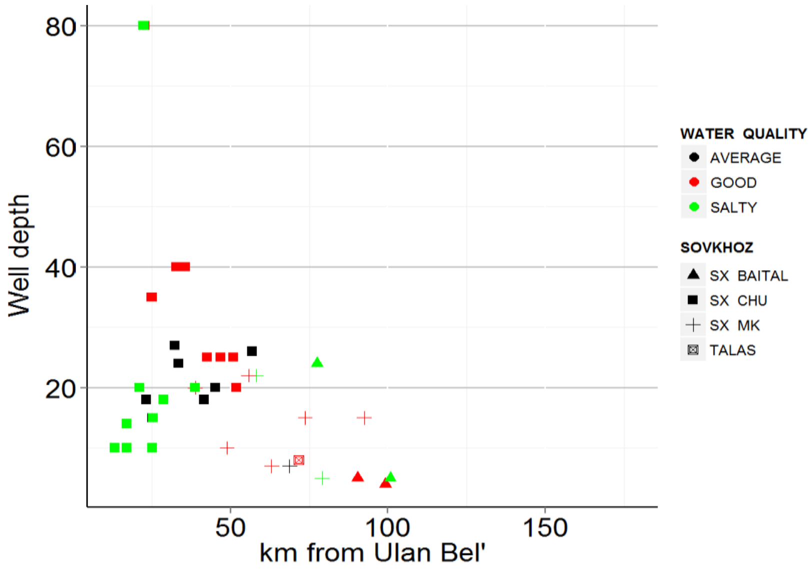

Figure S3 shows that, for a livestock owner based at Ulan bel', although most nearby wells are relatively shallow, many of them are of poor quality. The wells with good quality water tend to be relatively deep. Further away, in former Moiynkum and Baital *sovkhoz* there are a number of shallow wells, which are of good or average quality. Some of these are occupied by owners based at Sary Ozek and Malye Kamkale; others are occupied by owners from Ulan bel' who have also tended to move greater distances in recent years. This sample of wells, representing those for which respondents had knowledge, thus appears to suggest the existence of a group of shallower wells to the south and east.

5. Snow cover gradients

Figure S4. Average percent snow cover 2001-2012

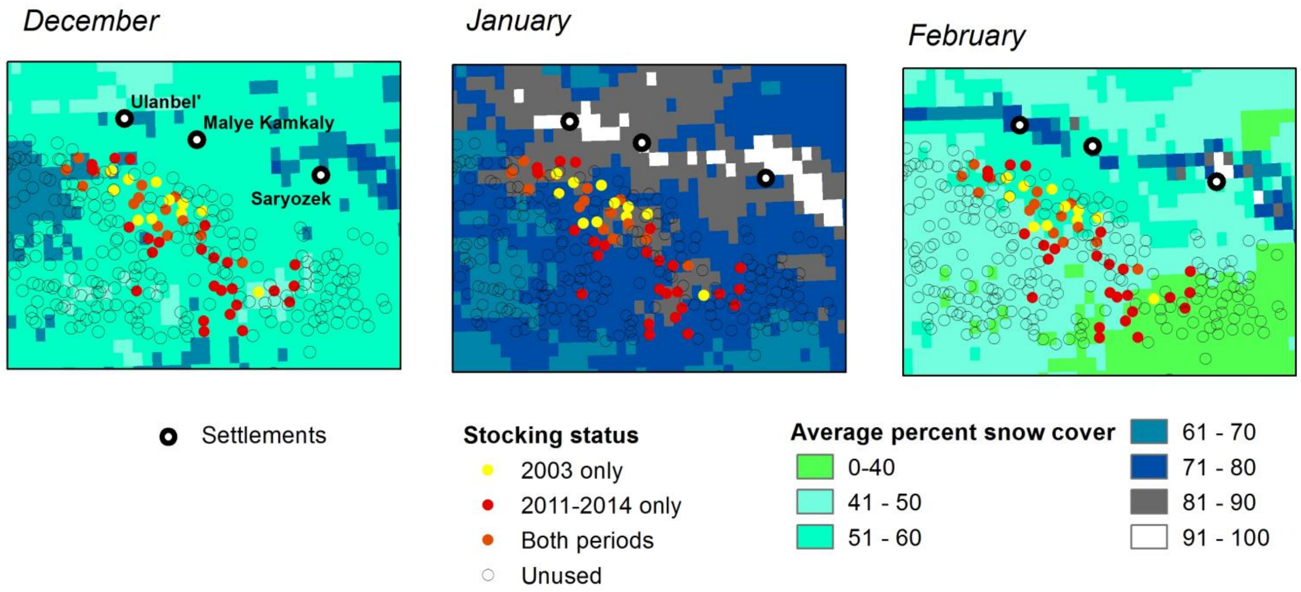

6. Water sources and relief

There are still some unoccupied areas, such as the large area to the south of Ulan bel' and in northern Talas raion where the large number of unused sites may require further explanation. We can see from Fig. 2 that the areas directly south of Ulan bel have low vegetation density, and Fig. S4 in supplementary materials suggests that snow cover there is higher than in eastern areas at the same latitude, yet such factors are rarely mentioned by users (Kerven et al., 2016). In some of these areas at least, water sources tend to be bore holes, which may be why they are little used, and livestock owners themselves also mentioned transport difficulties related to sand depth and dune height. Despite the convenient metalled road leading to the south, dunes are perpendicular to the direction of travel into the desert, dunes are higher and the sand deeper, making wells in these areas difficult to access (Kerven et al., 2016). Both bore hole distribution and relief are visible on the elevation image shown in Fig. S5, in which the orientation and amplitude of dunes to the south west of former sovkhoz Chu and the far south of Baital are visible.

Figure S5. Dune relief and well locations in the Moiynkum desert, with water sources for former sovkhoz Chu. Source: Advanced Spaceborne Thermal Emission and Reflection Radiometer (ASTER) Global DEM dataset (ASTGTM) is a product of METI and NASA.

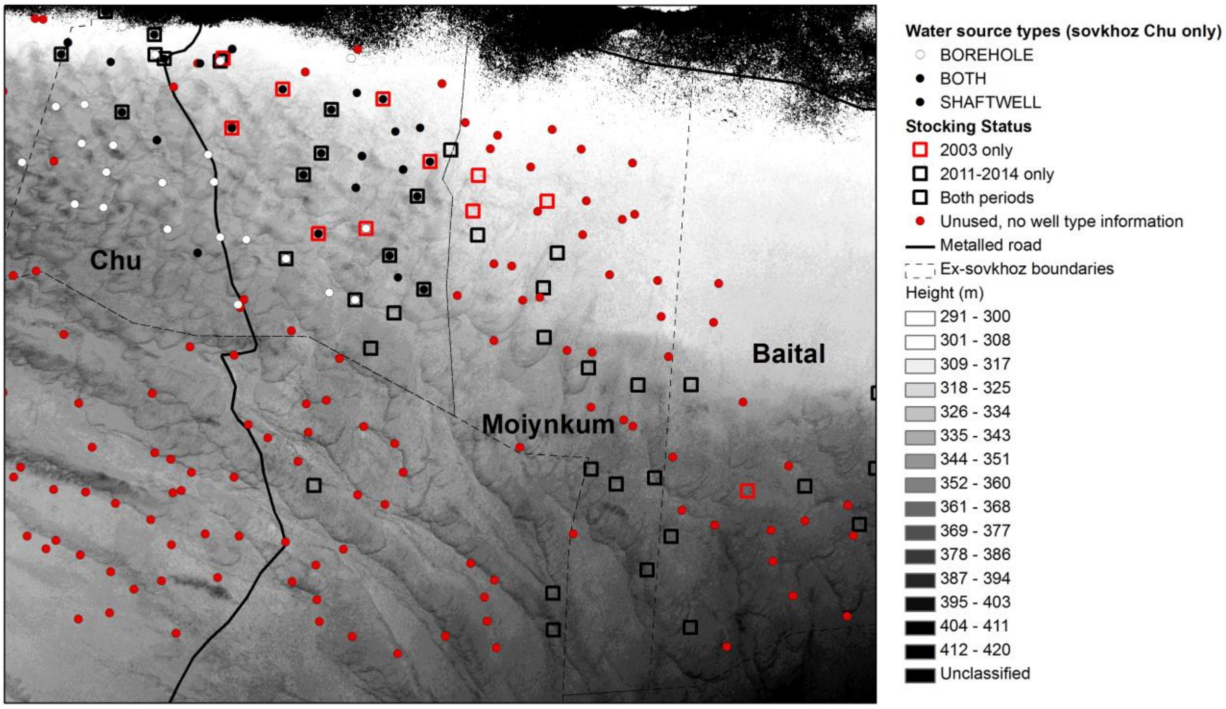

7. Snow cover and sequencing of occupation

Figure S6. Mean winter snow cover at sites used in 2003 alone, 2011-2014 alone, both periods and unused sites across the Moiynkum desert (n= 11, 25,15,231). Mean snow cover at sites used in 2002-3 alone and those used between 2011 and 2014 is 39.7% and 37.7% respectively (difference is significant: t=2.86, df=25.238, p<0.005).

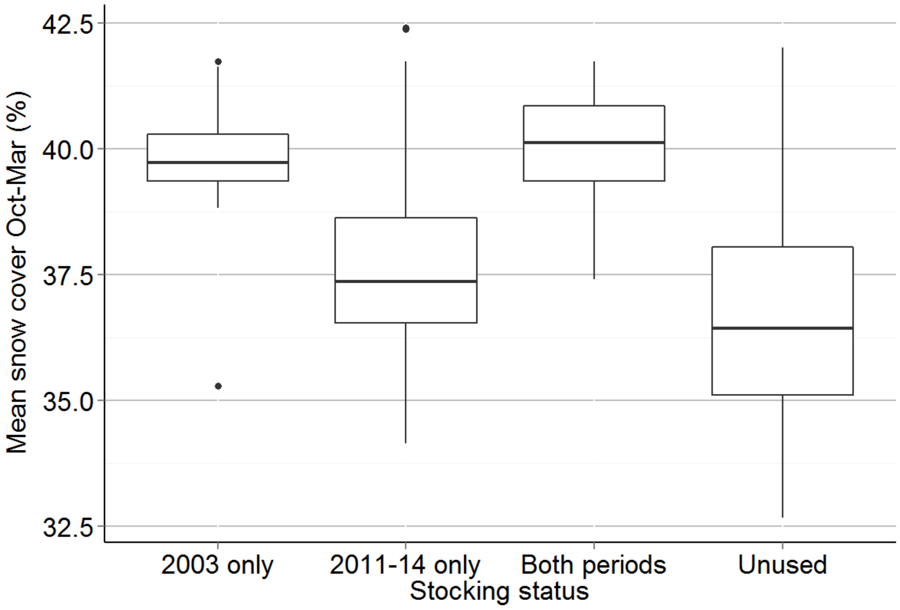

References

Hall, D. K., Salomonson, V. V. & Riggs, G. A. 2006. MODIS/Terra Snow Cover Monthly L3 Global 0.05Deg CMG. Version 5. [2001-2012].

Hird, J. N. & McDermid, G. J. 2009. Noise reduction of NDVI time series: An empirical comparison of selected techniques. *Remote Sensing of Environment,* 113**,** 248-258.

Kerven, C., Robinson, S., Behnke, R., Kushenov, K. & Milner-Gulland, E. J. 2016. A Pastoral frontier: from chaos to capitalism and the recolonisation of the Kazakh rangelands. *Journal of Arid Environments,* 127**,** 106-119.

Ministry of Agriculture of the Kazakh SSR 1969. *Instructions and Methodologies for Assessment of Hayfields and Pastures in the Territory of the Kazakh SSR,* Almaty.

Robinson, S., Kerven, C., Behnke, R., Kashenov, K. & Milner-Gulland, E. J. 2016. The changing role of bio-physical and socio-economic drivers in determining livestock distributions: a historical perspective from Kazakhstan. *Agricultural Systems,* 143**,** 169-182.
